# Hemichorea secondary to isolated temporal infarction with severe middle cerebral artery stenosis: a case report and review of literature

**DOI:** 10.1186/s12883-023-03230-6

**Published:** 2023-05-08

**Authors:** Hanrong Dong, Jingmin Zhao, Kwee-Yum Lee, Guangxun Shen

**Affiliations:** 1 Department of Neurology, The Third Bethune Hospital of Jilin University, Changchun, China; 2 Department of Orthopaedic Surgery and Musculoskeletal Medicine, University of Otago, Dunedin, New Zealand

**Keywords:** Hemichorea, Temporal Stroke, Cortical Infarction

## Abstract

**Background:**

Hemichorea typically results from a contralateral subthalamic nuclei (STN) lesion, although it has been reported in the cortex in a minority of cases. However, to our best knowledge, there are no documented cases in literature of hemichorea occurring as a secondary condition to an isolated temporal stroke.

**Case presentation:**

We present a case of an elderly female who sustained a sudden onset of hemichorea in her right extremities, predominantly in the distal region, lasting over a period of two days. Brain diffuse weighted image (DWI) demonstrated a high signal in the temporal region, while magnetic resonance angiography (MRA) revealed severe stenosis of the middle cerebral artery. During the symptomatic phase, computed tomography perfusion (CTP) revealed delayed perfusion in the left middle cerebral artery territory, characterized by the time-to-peak (TTP) measure. Based on the results of her medical history and laboratory tests, we were able to rule out the possibility of infectious, toxic, or metabolic encephalopathy. Her symptoms gradually improved with antithrombotic and symptomatic treatment.

**Conclusions:**

It is important to recognize and consider acute onset hemichorea as an initial symptom of stroke to avoid misdiagnosis and delays in appropriate treatment. Further research on temporal lesion that lead to hemichorea is warranted to gain a better understanding of the underlying mechanisms.

**Supplementary Information:**

The online version contains supplementary material available at 10.1186/s12883-023-03230-6.

## Background

Hemichorea is defined as a syndrome characterized by continuous, irregular, and involuntary jerky movements on one side of the body. Its pathogenesis can originate from infections, immune reaction, metabolic abnormalities, malignancy, neurodegeneration, vascular diseases, or drugs [1]. Hemichorea typically results from a focal lesion of the contralateral subthalamic nuclus (STN), which is thought to be an essential node in the complex neuronal network consisting of the basal ganglia and different motor cortical areas producing hyperkinetic movements, while causative lesions in cortex have been documented in a minority. However, to the best of our knowledge, there have been no previous reports in the literature of hemichorea resulting from isolated temporal stroke.

Herein, we present a case of an acute temporal stroke responsible for hemichorea in the right extremities, which have significantly resolved after treatment.

## Case presentation

**Fig. 1:**
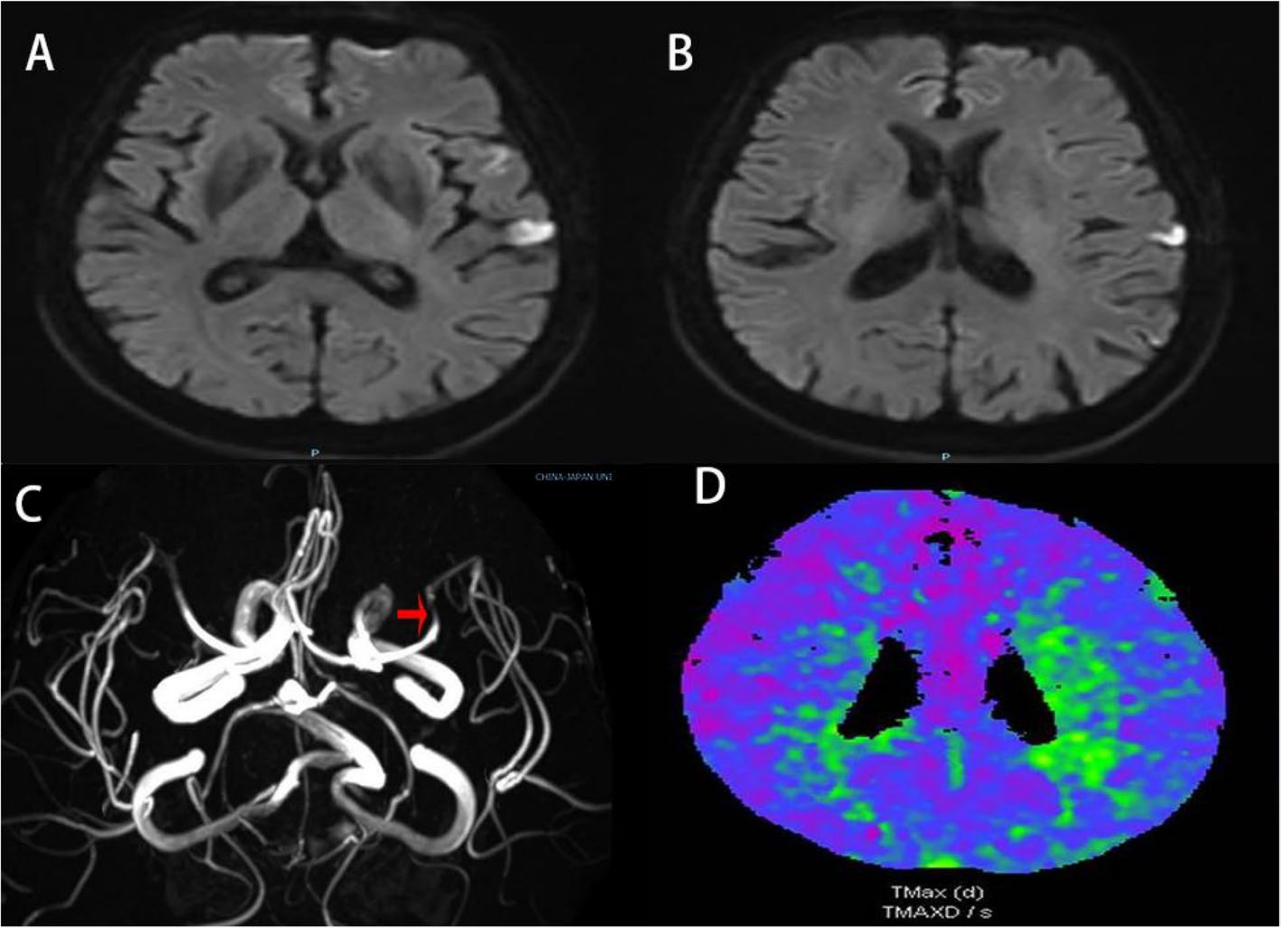
Brain images. DWI demonstrated high signal in the temporal region (**A** and **B**); MRA illustrated severe left M1 stenosis (arrow) (**C**); CTP during the symptomatic phase showed delayed perfusion in the left parietal cortex and corona radiata in the TTP measure (**D**). DWI = diffuse weighted image; MRA = magnetic resonance angiography; CTP = computed tomography perfusion; TTP = time-to-peak

A 71-year-old female with a past medical history of hypertension was admitted to our hospital due to hyperkinesia in her right extremities for two days. She had a sudden onset of involuntary flailing movements of her right upper extremity, unexpected grasping of her right hand, and abrupt kicks with her right lower extremity. These involuntary movements in the right upper and lower extremities were persistent, unsynchronized, variable and worsened when intentionally carrying out tasks (See Video 1 in the online supplementary material). The patient had mild dysarthria throughout the entire course of the illness, which abruptly deteriorated to complete aphasia with a decrease in muscle strength of her right upper extremity to level 3 for a duration of 10 min—during this time, her hemichorea continued. Her speech and muscle strength subsequently returned to normal, and she did not experience any further episodes. The results of a brain computed tomography (CT) scan showed unremarkable findings. Laboratory examinations and her medical history ruled out infectious, toxic, and metabolic causes of encephalopathy. Brain magnetic resonance images (MRI) illustrated an acute ischemic lesion in the left temporal cortex (Fig. 1A and B), and magnetic resonance angiography (MRA) of the head demonstrated severe stenosis in the left M1 branch (Fig. 1C). The computed tomography perfusion (CTP) scan during the symptomatic phase showed delayed perfusion in the left parietal cortex and corona radiata in the time-to-peak (TTP) measure (Fig. 1D).

The patient was given aspirin and atorvastatin separately, with a dosage of 100 mg and 20 mg, respectively, once a day and received 100 ml of butylphthalide injection twice daily. Despite being prescribed oral haloperidol at a dose of 2 mg twice daily for 3 days, she did not experience any relief of symptoms. Given no improvement, the dose of haloperidol was up-titrated to three times daily—4 mg in the morning, 2 mg in the afternoon, and 2 mg in the evening. Ten days after the initiation of antithrombotic therapy and pharmacological management of movement disorders mentioned above, she was discharged with significant improvement in hemichorea. The patient provided informed consent and has agreed to the publication of this case report.

## Discussion

Hemichorea is a rare initial or sole sign of acute ischemic stroke that originates purely from the cortex, especially in the temporal lobe, which is not typically associated with motor function. In this case report, we demonstrated a patient who exhibited hemichorea in the right extremities as the initial symptom of an isolated temporal stroke with severe middle cerebral artery stenosis, and no other intracranial pathology was found to account for the symptoms. This was confirmed by DWI and CTP imaging performed during the symptomatic phase. Furthermore, hemichorea was diagnosed, as opposed to stereotypy, due to the patient’s presentation of ongoing random-appearing sequences of one or more discrete involuntary movements or movement fragments. In contrast, stereotypies are repetitive, simple, rhythmic movements that can be voluntarily suppressed (e.g., simple back-and-forth movements such as waving or flapping the hands or arms) and they do not typically involve more complex sequences or movement fragments. There is probably no premonitory urge to move, and the movements tend to occur when the patient is stressed, excited, distracted or engrossed. Stereotypies can be stopped by distraction or initiation of another activity. In conclusion, stereotypy is distinguished from chorea by the predictability of the phenomenology and triggers of the movement [2].

By using search terms with Boolean operators—i.e., (“hemichorea” [Title/Abstract] OR “hemiballism” [Title/Abstract]) AND (“cortex” [Title/Abstract] OR “cortical” [Title/Abstract]), we searched the PubMed database up until March 2022 and identified 60 articles. Among those, 10 articles were selected which reported on hemichorea following a cortical lesion [3-12] (see Table 1). Of the 10 articles, only 5 reported on hemichorea after lesions that included the temporal lobe [3, 4, 7, 11 and 12], but none of which were purely secondary to a temporal lesion. Notably, symptoms arising from the cortex were less severe and had good long-term prognosis, as the symptoms subsided with treatment or resolved completely over time.

**Table 1:** Summary of cases of hemichorea following cortical lesions

Ref	Author	Year	HC/HB	lesions	pathogenesis
1	Carbayo á[3]	2020	L	R parietal and posterior frontal cortices	R M3 occlusion
			R	L insular and parietal cortex	L M2 branch occlusion
			L	R parietal and insular cortex	AF
			R	L insular, temporal, and parietal cortex	L M2 occlusion
2	Hernandez Fustes OJ[4]	2020	L	R temporo−parietal cortex	AF
3	Cotroneo M[5]	2019	L	R frontoparietal region	R carotid stenosis
			L	R frontoparietal and insular cortex	AF
			R	R frontal−parietal−insular cortex	AF
4	Strauss S[6]	2019	R	R parieto−occipital region	Paroxysmal AF
5	Jacob S[7]	2016	L	R mesial−temporal and hippocampal cortical regions, and minimally the occipital cortex	P2 focal occlusion
6	Shrestha P[8]	2015	R	L posterior parietal lobe	stenosis of the R−P1 segment
7	Hao M[9]	2015	L	both sides of the corona radiate and parietal cortex	stenosis on both of middle and posterior occlusion of ACA
8	Pichierri A[10]	2012	L	R premotor cortex area	tumor
9	Chung SJ[11]	2004	R	L parietal cortex	L M1 stenosis
			L	R insular cortex	NA
			L	R frontal, and insular cortex	NA
			L	R frontal, and parietal cortex	NA
			L	R parietal and temporal cortex	R ICA occlusion
			R	L temporal, insular cortex	L M1 occlusion
10	Krauss JK[12]	1999	R	L temporooccipital cortex	AVM

^*H*^^*C/HB* Hemichorea/hemiballism, *R* Right; *L* Left; *AF* Atrial fibrillation, *ACA* Anterior cerebral artery, *ICA* Internal carotid artery, *NA* Not available, *AVM* Arteriovenous malformation^

The mechanism accounting for this phenomenon may be attributed to the complexity and wide distribution of motor pathways involved in hemichorea, beyond the classic model of basal ganglia circuitry. Notably, the temporal lobe is likely to play a significant node in these pathways. When the metabolism of the temporal lobe is disrupted, it may weaken the inhibitory effect of the cortex, alter sensorimotor integration and spatial firing patterns, and ultimately result in subthalamic hyperactivity that manifests as hemichorea [13]. The hypothesis is strongly supported by the effective relief of symptoms observed in our case after an increased dosage of haloperidol.

During the symptomatic phase in this case, CTP showed hypoperfusion in the left parietal cortex and corona radiata, primarily due to severe MCA stenosis [14], which could have disturbed the metabolism of the basal ganglia, particularly in the striatum. The striatum, which is a part of the basal ganglia along with the thalamus, lentiform nucleus, and sensorimotor cortex, plays a crucial role in mediating muscle tone. The sudden onset of a transient ischemic attack, likely caused by hemodynamic compromise in MCA area, strongly supports this theory.

Most patients with hemichorea associated with a pure cortical infarct show a good prognosis as symptoms tend to disappear spontaneously or with medication, possibly owing to the structural integrity of the STN. Our patient’s hemichorea also demonstrated substantial improvement after 10 days of pharmacological treatment.

## Conclusion

Our case suggests that isolated temporal stroke caused by severe MCA stenosis can be associated with hemichorea in the absence of any other lesions. Therefore, clinicians should be aware that acute onset hemichorea could be an initial manifestation of acute ischemic stroke, to prevent misdiagnosis and delay in administering appropriate treatment. Further research on temporal stoke leading to hemichorea is necessary to better elucidate the underlying mechanisms.

## Supplementary Information

**Additional file 1.**

## Data Availability

All data and material supporting our findings are contained within the manuscript.

## Abbreviations

STN: Subthalamic nucleus
CT: Computed tomography
MRI: Magnetic resonance imaging
CTP: Computed tomography perfusion
TTP: Time-to-peak
DWI: Diffuse weighted imaging
MRA: Magnetic resonance angiography
